# CXCR-4 expression by circulating endothelial progenitor cells and SDF-1 serum levels are elevated in septic patients

**DOI:** 10.1186/s12950-018-0186-7

**Published:** 2018-05-16

**Authors:** Christian Patry, Daniela Stamm, Christian Betzen, Burkhard Tönshoff, Benito A. Yard, Grietje Ch. Beck, Neysan Rafat

**Affiliations:** 1 Department of Pediatrics I, University Children’s Hospital Heidelberg, Im Neuenheimer Feld 430, 69120 Heidelberg, Germany; 2 Institute of Physiology and Pathophysiology, Division of Cardiovascular Physiology, University of Heidelberg, Im Neuenheimer Feld 326, 69120 Heidelberg, Germany; 3 Department of Anaesthesiology and Critical Care Medicine, University Medical Center Mannheim, Theodor-Kutzer-Ufer 1-3, 68167 Mannheim, Germany; 4 Department of Medicine V, University Medical Centre Mannheim, Theodor-Kutzer-Ufer 1-3, 68167 Mannheim, Germany; 5 Department of Anaesthesiology and Critical Care Medicine, HELIOS Dr. Horst Schmidt Kliniken, Wiesbaden, Ludwig-Erhard-Straße 100, 65199 Wiesbaden, Germany; 6 Department of Neonatology, University Children’s Hospital Mannheim, University of Heidelberg, Theodor-Kutzer-Ufer 1-3, 68167 Mannheim, Germany; 7 Department of Pharmaceutical Sciences, Bahá’í Institute of Higher Education (BIHE), Teheran, Iran

**Keywords:** Endothelial progenitor cells, Homing, Sepsis, Chemokine receptors

## Abstract

**Background:**

Endothelial progenitor cell (EPC) numbers are increased in septic patients and correlate with survival. In this study, we investigated, whether surface expression of chemokine receptors and other receptors important for EPC homing is upregulated by EPC from septic patients and if this is associated with clinical outcome.

**Methods:**

Peripheral blood mononuclear cells from septic patients (*n* = 30), ICU control patients (*n* = 11) and healthy volunteers (*n* = 15) were isolated by Ficoll density gradient centrifugation. FACS-analysis was used to measure the expression of the CXC motif chemokine receptors (CXCR)-2 and − 4, the receptor for advanced glycation endproducts (RAGE) and the stem cell factor receptor c-Kit. Disease severity was assessed via the Simplified Acute Physiology Score (SAPS) II. The serum concentrations of vascular endothelial growth factor (VEGF), stromal cell-derived factor (SDF)-1α and angiopoietin (Ang)-2 were determined with Enzyme linked Immunosorbent Assays.

**Results:**

EPC from septic patients expressed significantly more CXCR-4, c-Kit and RAGE compared to controls and were associated with survival-probability. Significantly higher serum concentrations of VEGF, SDF-1α and Ang-2 were found in septic patients. SDF-1α showed a significant association with survival.

**Conclusions:**

Our data suggest that SDF-1α and CXCR-4 signaling could play a crucial role in EPC homing in the course of sepsis.

**Electronic supplementary material:**

The online version of this article (10.1186/s12950-018-0186-7) contains supplementary material, which is available to authorized users.

## Background

Endothelial barrier damage and dysfunction are core elements of sepsis pathophysiology. Without rapid restoration of endothelial cell function, septic patients will inevitably develop irreversible multi organ failure [1, 2]. In that respect, endothelial progenitor cells (EPC) might constitute a potential targeted treatment option in the future. It could be demonstrated, that EPC have the potential to regenerate and reconstitute damaged endothelial layers in several diseases like sepsis and acute respiratory distress syndrome (ARDS) [3–7]. We and others furthermore showed, that EPC in septic patients are distinctly mobilized and that elevated EPC levels in the circulation significantly correlate with survival in the course of sepsis [7–10]. However, the molecular pathways that underlie EPC mediated endothelial barrier regeneration in sepsis are still not well understood.

Endothelial progenitor cells are able to migrate into damaged subendothelial layers, subsequently promote angiogenesis and induce endothelial barrier regeneration, especially in states of systemic endothelial inflammation [3]. This sequence must be preceded by a directed EPC homing process [11]. The principal mechanisms of cellular homing to endothelial sites of inflammation are currently best examined in leukocytes [12] and it can be assumed, that similar mechanisms also influence EPC homing. Similar to leukocyte homing EPC homing is a coordinated multi-step-process including mobilization, chemotaxis and attachment [13], and its efficiency is influenced by the repertoire and the level of chemokine expression by the target tissue as well as the expression of the respective receptors on EPC [14–21] Molecular regulators that affect both leukocyte and EPC migration and activity are known and might also play a potential role in EPC homing especially in sepsis. The functional CXC-motive-chemokine receptor 4 (CXCR4) and its ligand stromal cell derived factor 1α (SDF-1α), the receptor for advanced glycation endproducts (RAGE), the cell-surface bound P-selectin ligand-1 (PSGL-1) and the CXC-motive-chemokine receptor 2 (CXCR-2) have been demonstrated to both impact leukocyte and EPC migration and homing processes [11, 22–30]. Furthermore, the tyrosine-kinase KIT (c-Kit) has been demonstrated to recruit endothelial progenitor cells to inflamed endothelium [31] and to modulate bone marrow derived progenitor cell mobilization [32]. Additionally, vascular endothelial growth factor (VEGF^9^) and Angiopoietin 2 (Ang2^10^), the traditional regulators of angiogenesis, are known EPC mobilizers and inductors of EPC migration [33–35].

These mediators and their receptors could be important promoters or inhibitors of the EPC homing process in sepsis and thereby influence the EPC mediated endothelial regeneration in systemic inflammation. Thus, we designed this clinical study, to primarily investigate changes in the expression of CXCR-4, CXCR-2, RAGE, c-Kit and PSGL-1 on EPC surfaces and to assess potential correlations with the serum levels of SDF-1α, VEGF and Ang2 in septic patients.

## Methods

### Subjects

In our study, we included over a 3-year period patients with sepsis from the ICU of the University Hospital Mannheim within 48 h after onset of sepsis or at admission to the intensive care unit. Included patients met the diagnostic criteria for sepsis according to the American College of Chest Physicians and the Society of Critical Care Medicine [36]. Disease severity was measured on the basis of the Simplified Acute Physiology Score II (SAPS II^11^) [37], and mortality was defined as death occurring within 28 days after diagnosis. Exclusion criteria were the use of statins or angiotensin-converting enzyme inhibitors, the use of activated protein c, the use hydrocortisone as well as cardiogenic or hemorrhagic shock, chronic obstructive pulmonary disease, isolated acute respiratory distress syndrome or the absence of mechanical ventilation. We furthermore included ICU control patients with need for mechanical ventilation as well as healthy controls, which were volunteers from the laboratory staff. ICU controls did not meet the criteria for sepsis, septic shock, or systemic inflammatory response syndrome. The study was approved by the Ethics Committee of the University of Heidelberg. Approved and written informed consent was obtained from all study subjects.

### Blood sampling

20 ml blood obtained from septic patients was collected within 24 h after sepsis onset. Blood from ICU control patients was collected within 24 h after admission to the ICU.

### Isolation of peripheral blood mononuclear cells

Ficoll gradient centrifugation (Amersham Biosciences, Freiburg, Germany) was used to isolate peripheral blood mononuclear cells (PBMC) from the peripheral blood of study subjects: Peripheral blood was diluted 1:2 with phosphate buffered saline and gently layered on top of the Ficoll solution. Centrifugation was performed at 20 °C with 400 g for 30 min. Then, the cells in the interphase were aspirated and centrifuged at 20 °C with 300 g for 15 min. The supernatant was discarded and the pellet incubated with erythrocyte lysis buffer for 8 min. After that, the cells were washed two times with phosphate buffered saline and centrifuged (20 °C with 300 g for 10 min). Thereafter, PBMC were prepared and analyzed by flow cytometry.

### Flow cytometry

The expression of cell-surface antigens was quantified by immunostaining as described previously [7]. We used the following monoclonal antibodies (anti-human): PE-conjugated CD133 (Miltenyi Biotec, Bergisch-Gladbach, Germany), PerCP-conjugated CD34 (BD Biosciences, Heidelberg, Germany), and either FITC-conjugated CXCR-4 or APC-conjugated c-Kit, APC-conjugated CXCR-2 (all R&D Systems, Wiesbaden-Nordenstadt, Germany), PE-conjugated PSGL1 (BD Biosciences, Heidelberg, Germany) or the indirect rabbit anti-human polyclonal RAGE antibody (Biozol, Eching, Germany), for which a FITC-conjugated anti-rabbit IgG antibody (Invitrogen, Karlsruhe, Germany) was used. We used a FACSCalibur flow cytometer (BD Biosciences) for flow cytometry. FACS-data analysis was performed with WinMDI 2.8 software (Scripps Research Institute, La Jolla, CA). EPC counts are expressed as percentage referred to total PMBC in each study subject.

### Enzyme-linked immunosorbent assay

VEGF, SDF-1α and Angiopoietin-2- serum concentrations were measured with enzyme linked immunosorbent assay kits in triplicate samples according to the instructions provided by the manufacturer (R&D Systems, Wiesbaden-Nordenstadt, Germany).

### Statistical analysis

All data were examined for normal and non-Gaussian distribution by the Kolmogorov-Smirnov test. Results are presented as mean ± SD (Standard-Deviation). Both parametric and nonparametric methods were used. For comparison among normally distributed groups, one-way ANOVA, followed by pairwise multiple comparison (Student-Newman-Keuls method) was used. For non-normally distributed data, the nonparametric Kruskal-Wallis test followed by an all pairwise multiple comparison (Dunnett’s method) was used. We predicted survival probability from EPC numbers based on logistic regression analysis. Pearson- Spearman correlation analyses were considered for all target variables. *P* < 0.05 is considered to be statistically significant. All analyses were performed using the SAS system (version 8.2).

## Results

### Patient population

In Table 1 relevant clinical data of the study patients with regards to age, gender, mortality, SAPSII score, type of infection, WBC count and PCT are summarized. There was a significant increase of PCT levels (20-fold) in sepsis patients compared to ICU patients (Table 1). No significant differences in WBC and SAPSII values were found between sepsis patients and ICU patients (Table 1). Between survivors and non-survivors of the sepsis group no significant differences in PCT levels, WBC and SAPSII values were found (Additional file 1: Table S1).

**Table 1:** Clinical characteristics of patients and controls

Characteristics	Healthy controls	ICU controls	Septic patients
number of subjects	15	11	30
Age (years)			
Mean ± SD	60,4 ± 14,3^a^	57,8 ± 14,2	35,8 ± 12,9
Gender			
Male (%)	3 (20)	6 (54,5)	20 (66,6)
Female (%)	12 (80)	5 (45,5)	10 (33,3)
Mortality < 28 days (%)		0 (0)	15 (50)
Mean SAPS II score (range)		38,8 (15–59)	49,9 (22–74)
Type of infection n (%)			
Pneumonia			7 (23)
Peritonitis			9 (30)
Meningitis			4 (13)
Pancreatitis			1 (3)
Gastrointestinal tract			3 (10)
Necrotic fasciitis			2 (7)
Cholangitis			2 (7)
Cholecystitis			1 (3)
Trauma			1 (3)
WBC (×10^9^/L)		10.5	15.5
PCT (ng/ml)		1,9	22,9^a^

Clinical data of study participants for age, gender, mortality, Simplified Acute Physiology Score (SAPS) II score, type of infection, white blood cell (WBC) count and procalcitonin (PCT) refer to the time point of blood sampling. ^a^The mean age in the group of healthy controls was significantly lower compared to the patient groups (*p* = 0.0001). There was no statistical difference in mean age between the two patient groups (*p* = 0,61). There was a significant difference in PCT levels between septic patients and ICU controls (*p* = 0,0002)

### Endothelial progenitor cells in septic patients and correlation with survival

**Fig. 1:**
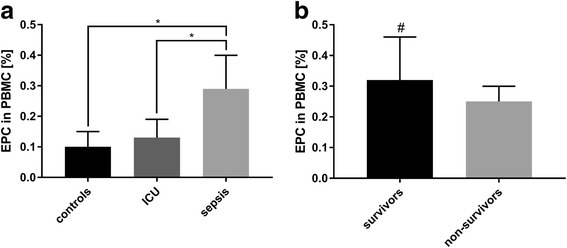
Numbers of circulating endothelial progenitor cells. **a** FACS analysis of CD34/CD133-positive cells in the peripheral blood mononuclear cell fraction of healthy volunteers (*n* = 15), non-septic intensive care unit (ICU) patients (*n* = 11) and septic patients (*n* = 30). Significant differences were found between the three groups. **b** FACS analysis of CD34/CD133-positive cells of septic patients, stratified for survival. Data are given as mean ± SEM; * marks a significant difference (*p* < 0,05), ^#^ marks a significant influence on EPC on survival-probability in logistic regression analysis including CXCR-4-expression as supressing variable (*p* = 0,037, odds ratio = 0,167)

The percentage of EPC was significantly increased by 120% in septic patients compared to ICU and by 190% compared to healthy controls, while the difference in EPC numbers between ICU and healthy controls was smaller and not significant (Fig. 1) Within the group of septic patients, sepsis survivors had increased numbers of EPC compared to non-survivors by 28% (Fig. 1). Logistic regression analysis revealed a significant influence of EPC number increase on survival probability (odds ratio: 0,17, *p* = 0,037).

### Chemokine and other receptors expressed by endothelial progenitor cells

**Fig. 2:**
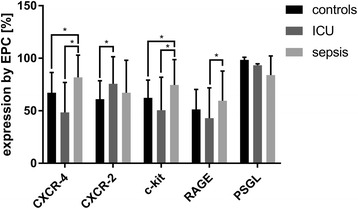
Upregulation of chemokine and other receptors by endothelial progenitor cells. FACS analysis of the of CXCR-4, CXCR-2, c-Kit, RAGE and PSGL-1 by CD34/CD133–positive cells in the peripheral blood mononuclear cell fraction of healthy volunteers (*n* = 15), non-septic intensive care unit (ICU) patients (*n* = 11) and septic patients (*n* = 30). * marks a significant difference (*p* < 0,05). *CXCR-4*, CXC-motive-chemokine receptor 4; *c-Kit*, tyrosine kinase KIT; *CXCR-2*, CXC-motive-chemokine receptor 2; *RAGE*, receptor for advanced glycation products; *PSGL-1*, P-selectin ligand 1

CXCR-4 expression on EPC from septic patients was significantly increased by 69% compared to ICU controls and by 22% compared to healthy controls (Fig. 2). In contrast, CXCR-2 expression on EPC from septic patients showed no significant difference compared to ICU- or healthy controls (Fig. 2). The expression of c-Kit on EPC from septic patients was significantly higher compared to ICU- (increase by 47%) or healthy controls (increase by 19%) (Fig. 2). The expression of RAGE on EPC from septic patients was significantly higher compared to ICU controls (increase by 38%) but not compared to healthy controls (Fig. 2). The expression of PSGL-1 on EPC from septic patients was comparable to ICU patients and healthy controls (Fig. 2). CXCR-4, c-Kit and RAGE expression in sepsis non-survivors was increased compared to survivors, but these results were not significant (Additional file 1: Figure S2). Logistic regression analysis revealed, that CXCR-4 expression by EPC increases the predictive value of EPC numbers on survival probability in logistic regression analysis.

### Serum concentration of the growth factors VEGF, SDF-1α and Ang-2 is elevated in septic patients

**Fig. 3:**
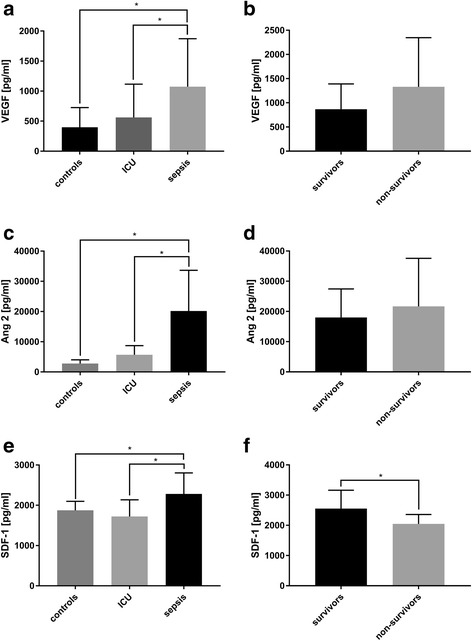
Upregulation of mobilizing growth factors in serum. **a** Vascular endothelial growth factor (VEGF), (**c**) angiopoietin (Ang)-2 and (**e**) stromal cell-derived factor 1α SDF-1α concentrations were detected in the serum of healthy volunteers (*n* = 15), non-septic intensive care unit (ICU) patients (*n* = 11) and septic patients (*n* = 30). **b**, **d**, **f** The group of septic patients was also divided by survival and serum concentrations of the three mobilizing factors are indicated. The results are expressed as ± SD; * marks a significant difference (*p* < 0,05)

**Fig. 4:**
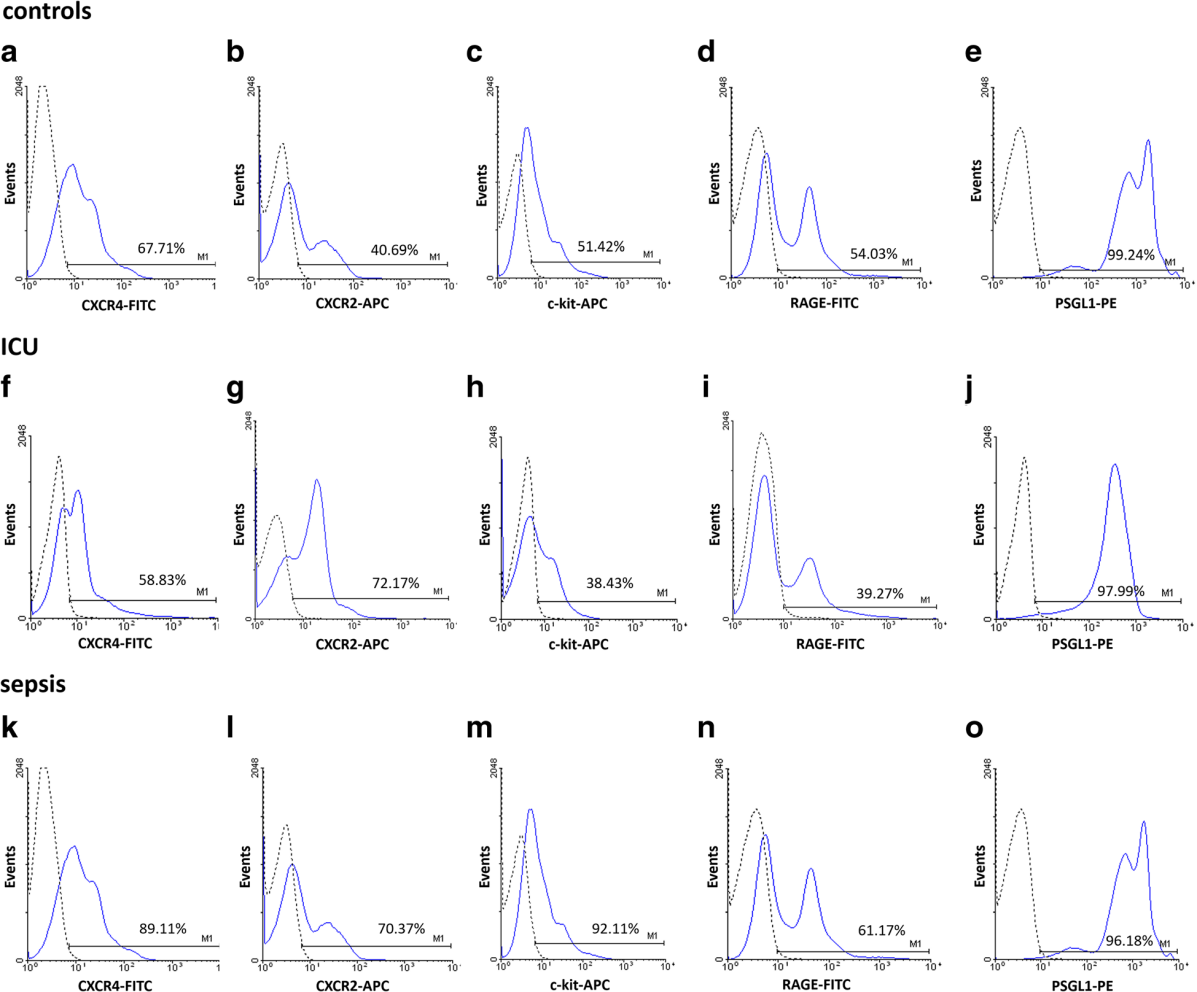
FACS analysis data representative for each investigated group: healthy volunteers (**a**-**e**), ICU controls (**f**-**j**) and septic patients (**k**-**o**); histograms show the percentage of CXCR-4, CXCR-2, c-Kit, RAGE and PSGL-1 expression in the population of CD34/CD133-positive cells. The dotted line in histograms represents the negative control. *APC*, allophycocyanin; *FITC*, fluorescein; *PE*, phycoerythrin; *CXCR-4*, CXC-motive-chemokine receptor 4; *c-Kit*, tyrosine kinase KIT; *CXCR-2*, CXC-motive-chemokine receptor 2; *RAGE*, receptor for advanced glycation products; *PSGL-1*, P-selectin ligand 1

Serum VEGF concentrations were significantly increased in septic patients by 73% compared to ICU controls and by 161% compared to healthy controls (Fig. 3a). Similarly, also SDF-1α and Ang-2 concentrations were significantly increased in septic patients compared to ICU and healthy controls (Fig. 3c & 3e). There was no significant difference in VEGF- and Ang2- serum concentrations between sepsis survivors and sepsis non-survivors (Fig. 3b & 3d). However, SDF-1α serum concentrations were significantly increased in sepsis survivors compared to non-survivors (by 25%) (Fig. 3f). For the entire study population, a significant correlation between EPC numbers and serum levels of VEGF (*r* = 0.21, *p* = 0.03), SDF-1α (*r* = 0.53, *p* = 0.001) and Ang-2 (*r* = 0.37, *p* = 0.0002) was observed. There was also a positive correlation between EPC number and VEGF serum levels in the ICU group (*r* = 0.42, *p* = 0.05) and the group of healthy controls (*r* = 0.46, *p* = 0.02), while the EPC concentration in septic patients or in the survivor/non-survivor subgroups was not correlated with serum VEGF, SDF-1α or Ang-2 (Fig. 4).

## Discussion

In this study we detected in septic patients an increase of circulating EPC which expressed significantly more CXCR-4, c-Kit and RAGE than EPC from non-septic patients. Furthermore, the serum levels of SDF-1α were significantly increased in both septic patients and survivors of sepsis. EPC numbers showed to be associated with sepsis survival probability. These findings indicate, that the SDF-1α/CXCR-4 signalling might be involved in EPC mediated regenerative processes during sepsis.

Several research groups including our own have demonstrated, that septic patients and animals exhibit increased levels of circulating EPC and that there is a positive correlation with survival. EPC numbers have been analysed and calculated in these studies using flowcytometry [7–10, 38]. However, in conflict with these findings, studies based on colony forming assays for EPC number analysis indicate, that EPC mobilization during sepsis is not enhanced [39, 40]. Essentially, the reasons behind these controversial findings still remain unresolved, but the differences in EPC purification- and measurement methodologies might play a role. In our study, we could again show a significant increase in EPC numbers in septic patients compared to ICU controls using flowcytometry. Thus, there are now multiple and independent results available, which indicate that sepsis leads to an increased mobilization of EPC into the peripheral blood. In addition, our study results revealed a positive influence of EPC numbers on sepsis survival probability in linear regression analysis. This result is also consistent with our previous findings and that of others [7, 41].

Both EPC mobilization and EPC homing to damaged endothelial layers are complex migratory processes, which involve several adhesion molecules, chemoattractants and respective receptors, like CXCR-4, CXCR-2, c-Kit, RAGE and PSGL-1 [14–21]. P-selectin glycoprotein ligand-1 (PSGL-1) signaling can increase the pro-angiogenic potential of EPC [14] and is involved in neutrophil recruitment in an abdominal sepsis model [27]. A downregulation of the CXC-motive-chemokine receptor-2 on neutrophils in severe sepsis impairs their migratory properties [29]. CXCR-2 is also involved EPC recruitment [30]. However, both PSGL-1 and CXCR-2 expression by EPC did not show significant differences in comparison to ICU controls in our study. On the contrary, we could demonstrate, that EPC from septic patients exhibit a significantly increased expression of the surface receptors CXCR-4, c-Kit and RAGE in comparison to EPC from ICU-controls and healthy controls. The expression of CXCR-4 was already shown to be increased on lymphocytes in sepsis [22] resulting in improved migration and activation. Levels of its ligand SDF-1α are also increased in septic states [23]. The SDF-1α /CXCR-4 axis is furthermore involved in EPC recruitment to the spleen [24] and CXCR-4 influences EPC homing through cellular polarization [11]. The receptor for advanced glycation endproducts RAGE is expressed by several cells of the innate immune system and activates NF-κ-B signaling [25]. RAGE signaling is also involved in integrin dependent homing of EPC [26]. The proto-oncogene c-Kit seems to play a crucial role in EPC recruitment to inflamed endothelium: EPC adhesion to tumor necrosis factor (TNF)-α treated endothelial cells mediated via c-Kit involves the intracellular Akt-pathway (Proteinkinase B, Akt) and can be prevented, when pretreating EPC with the c-Kit inhibitor imatinib [31]. Thus, the upregulation of CXCR-4, c-Kit and RAGE by EPC shown in our study indicates, that these factors could be important mediators of EPC homing in sepsis. But CXCR-2 and PSGL-1 might rather play minor roles in that respect.

Associated with the increased CXCR4 expression by EPC from septic patients in our study, we could also detect increased serum levels of the CXCR-4 ligand SDF-1α in septic patients. This finding is consistent with previous publications [42]. Furthermore, we were also able to show, that SDF-1α serum levels were significantly higher in sepsis survivors compared to non-survivors. Since the SDF-1α/CXCR-4-signalling axis impacts EPC recruitment to peripheral tissues, according to our results it could also be involved in promoting EPC homing in sepsis and thereby promote endothelial layer regeneration. In support of this Fan et al. found, that the synergistic application of both VEGF and SDF-1α leads to an increase of circulating EPC numbers and increased survival in septic rats [43]. The application of CTCE-0214, a SDF-1α peptide analog and CXCR-4 agonist, significantly suppressed TNF and interleukin (IL)-10 concentrations and improved survival in murine systemic inflammation [44] and sepsis [43].

Besides SDF-1α, we could also detect an increase of VEGF and Ang2-serum levels in septic patients. A positive correlation of VEGF, Ang2 and SDF-1α with EPC levels in septic patients compared to controls in our study indicates an impact of those factors on mobilization of EPC from the bone marrow during sepsis as shown before [34, 45–47].

Our study underlies the limitation that there is currently no unique single surface marker identified to clearly detect and isolate the EPC phenotype when using flowcytometry. However, culture based EPC purification methods, even if they are simple to perform, often yield heterogeneous cell populations, when analyzing surface marker distributions with flowcytometry afterwards [48]. Via using the progenitor cell marker CD133 in our FACS based EPC analysis, we could exclude mature endothelial cells from EPC counting. However, our EPC population counts likely include small amounts of hematopoietic stem cells, since the classical definition of EPC requires an endothelial marker protein like VEGF-R2 or CD31. Another limitation or our study arises from its cross-sectional design, resulting in a lack of information on EPC number changes or changes in surface receptor expressions by EPC in the disease course of sepsis.

## Conclusions

In conclusion, we have demonstrated here for the first time that EPC in the clinical setting of sepsis exhibit a high expression of CXCR-4, RAGE and c-Kit as potential promoters of EPC homing. In concert with that the serum level increase of the CXCR4-ligand SDF-1α was closely associated with sepsis survival, as were EPC numbers. Thus, our study provides first indications, that the SDF-1α/CXCR-4 signalling axis might be involved in EPC homing to damaged endothelial layers in sepsis, which is the prerequisite step for further EPC based regeneration processes. RAGE and c-Kit may also play distinct roles in that respect. Further studies will have to be performed to increase our understanding of the molecular pathways underlying EPC based barrier regeneration in sepsis in order to derive new targeted therapy options in the future.

## Additional file

Additional file 1: **Figure S1**: FACS analysis data representative for each investigated group. **Figure S2**: Upregulation of chemokine and other receptors by endothelial progenitor cells in survivors and non-survivors of sepsis. **Table S1**: Clinical characteristics of sepsis survivors and non-survivors. (DOCX 1076 kb)

## Abbreviations

Ang2: Angiopoietin 2
ARDS: Acute Respiratory Distress Syndrome
c-Kit: Tyrosine kinase KIT
CXCR-2: CXC-motive-chemokine receptor 2
CXCR-4: CXC-motive-chemokine receptor 4
EPC: Endothelial progenitor cells
PSGL-1: P-selectin ligand 1
RAGE: Receptor for advanced glycation products
SAPSII: Simplified Acute Physiology Score II
SDF-1α: Stromal cell derived factor 1α
VEGF: Vascular endothelial growth factor
