# Supplementary material for: CXCR-4 expression by circulating endothelial progenitor cells and SDF-1 serum levels are elevated in septic patients
Source: J Inflamm (Lond). 2018 May 16;15:10. doi: 10.1186/s12950-018-0186-7 (PMC5956812; doi:10.1186/s12950-018-0186-7)
Supplement: Supplementary file 1 — Figure S1: FACS analysis data representative for each investigated group. Figure S2: Upregulation of chemokine and other receptors by endothelial progenitor cells in survivors and non-survivors of sepsis. Table S1: Clinical characteristics of sepsis survivors and non-survivors. (DOCX 1076 kb) [file 12950_2018_186_MOESM1_ESM.docx]

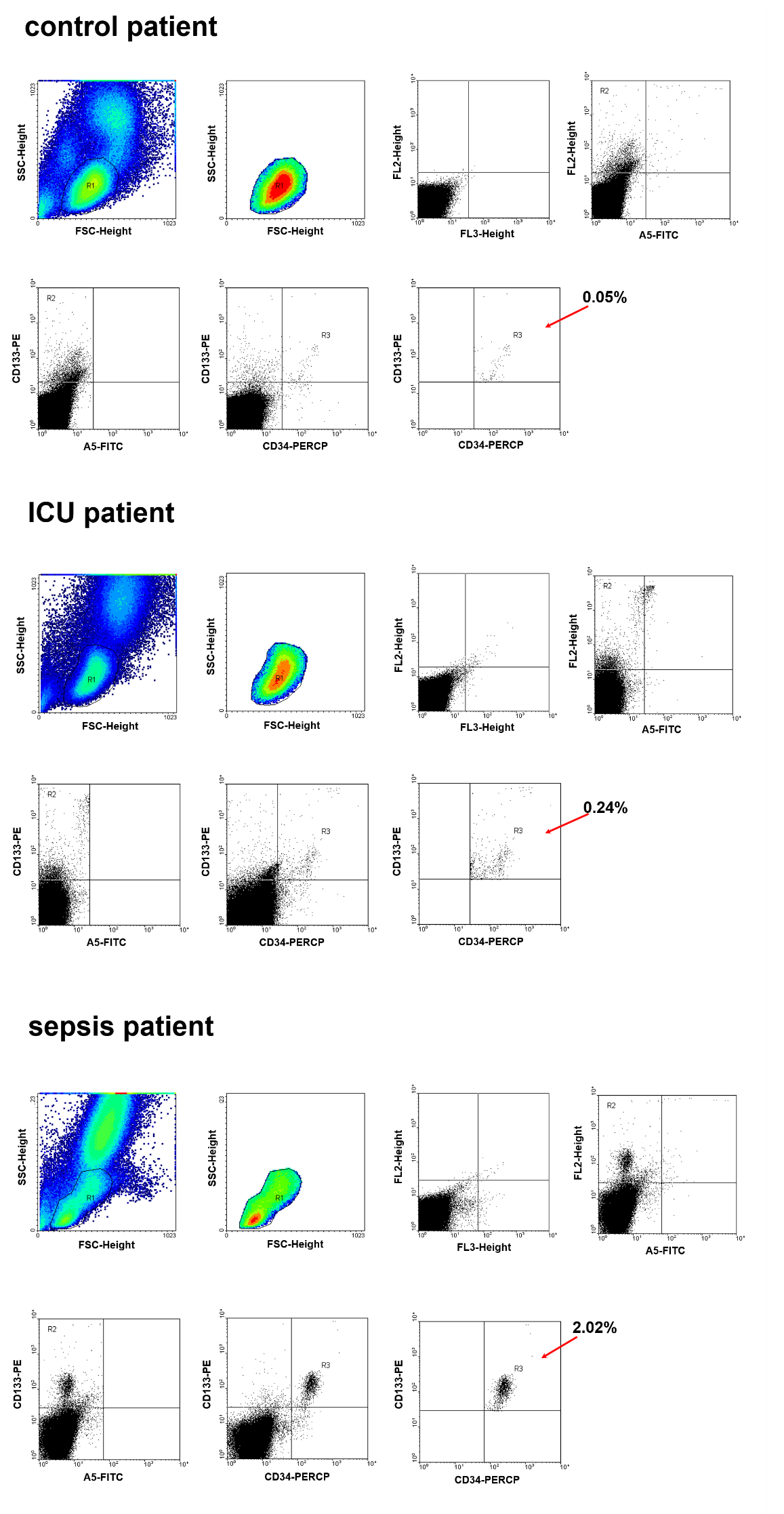
**Suppl. Figure 1**

**FACS analysis data representative for each investigated group**: control, -ICU and septic patient; histograms show the distribution of CD34/CD133-positive cells in FACS analysis. *APC*, allophycocyanin; *FITC*, fluorescein; *PE*, phycoerythrin.

**Supplemental Figure 2**


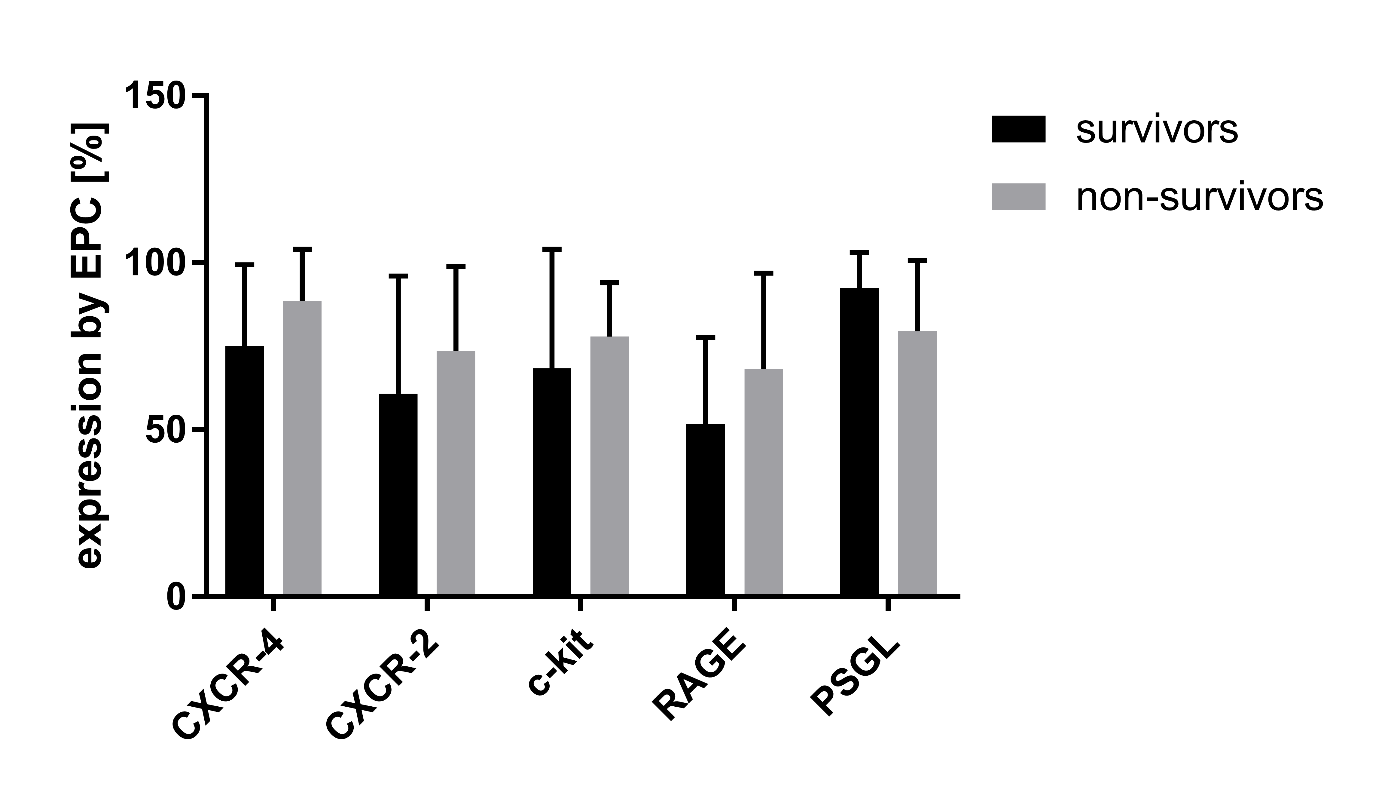


**Upregulation of chemokine and other receptors by endothelial progenitor cells in survivors and non-survivors of sepsis.** FACS analysis of CXCR-4, CXCR-2, c-Kit, RAGE and PSGL-1 by CD34/CD133–positive cells in the peripheral blood mononuclear cell fraction of survivors (n=15) and non-survivors (n=15) of sepsis. * marks a significant difference (p<0,05).

*CXCR-4*, CXC-motive-chemokine receptor 4; *c-Kit*, tyrosine kinase KIT; *CXCR-2*, CXC-motive-chemokine receptor 2; *RAGE*, receptor for advanced glycation products; *PSGL-1*, P-selectin ligand 1

**Supplemental Table 1**

**Clinical characteristics of sepsis survivors and non-survivors**

Clinical data of sepsis survivors and non-survivors for age, Simplified Acute Physiology Score (SAPS) II score, white blood cell count (WBC) and procalcitonin (PCT) refer to the time point of blood sampling. There was no significant difference in PCT levels, WBC and SAPSII values between survivors and non-survivors.

| characteristics | survivors | non-survivors |
| --- | --- | --- |
| number of subjects | 15 | 15 |
|  |  |  |
| Age (years) |  |  |
| *mean ± SD* | 58,4 ± 12,9 | 62,4 ± 13,8 |
|  |  |  |
| mean SAPS II score (range) | 49,9 (22 – 74) | 50,0 (26 – 73) |
|  |  |  |
| WBC (x10^9^/L) | 12,9 ± 9,4 | 18,0 ± 12,2 |
| PCT (ng/ml) | 19,0 ± 24,5 | 26,9 ± 42,5 |
